# The C-MAC videolaryngoscope compared with conventional laryngoscopy for rapid sequence intubation at the emergency department: study protocol

**DOI:** 10.1186/s13049-015-0119-x

**Published:** 2015-04-24

**Authors:** Simon Sulser, Dirk Ubmann, Martin Brueesch, Georg Goliasch, Burkhardt Seifert, Donat R Spahn, Kurt Ruetzler

**Affiliations:** Institute of Anaesthesiology, University and University Hospital Zurich, Zurich, Switzerland; Department of Cardiology, Medical University Vienna, Vienna, Austria; Epidemiology, Biostatistics and Prevention Institute, Department of Biostatistics, University of Zurich, Zurich, Switzerland; Outcomes Research Consortium, Cleveland, Ohio USA

**Keywords:** Emergency airway management, Tracheal intubation, Rapid sequence induction

## Abstract

**Background:**

Especially in the emergency setting, rapid and successful airway management is of major importance. Conventional endotracheal intubation is challenging and requires high level of individual skills and experience. Videolaryngoscopes like the C-MAC are likely to offer better glottis visualization and serve as alternatives to conventional endotracheal intubation. The aim of this study is to compare clinical performance and feasibility of the C-MAC videolaryngoscope compared to conventional endotracheal intubation in the emergency setting.

**Methods/Design:**

This study is designed as a prospective, patient-blinded, mono-center, randomized cohort study. This study will be performed at the Emergency Department of the University Hospital Zurich, Zurich, Switzerland. All patients transferred to the Emergency Department and requiring emergent endotracheal intubation will be screened. Successful intubation with first intubation attempt will serve as the primary outcome. Time to intubation, intubation attempts, Cormack & Lehane Score, ease of intubation, complications, necessity of using alternate intubation device, maximum drop of saturation, and potential technical problems serve as secondary outcomes.

**Discussion:**

In the clinical setting, the ultimate success rate of endotracheal intubation ranges between 97% and 99%. Unexpected difficulties during laryngoscopy and poor glottis visualization occur in up to 9% of all cases. In these cases, videolaryngoscopes may increase success rate of initial intubation attempt and thereby patient safety.

**Trial registration:**

www.clinicaltrials.gov (identifier NCT02297113).

## Background

Successful and rapid airway management is of major importance in the emergency setting and in specific situations life-saving. Beside a high level of expertise along with regular training and practice, successful endotracheal intubation sometimes requires additional tools to assist tracheal intubation [1-3].

Rapid Sequence Intubation (RSI) is indicated in all patients who are considered not sober and/or have an increased risk of gastric regurgitation and aspiration [4]. The main objective of this technique is to minimize the time interval between loss of protective airway reflexes and tracheal intubation with a cuffed endotracheal tube. This period is most critical, since aspiration of gastric content may to occur [5]. In the emergency setting, all patients are strictly considered not sober and RSI is obligatory indicated.

Videolaryngoscopy has been introduced to allow monitoring of conventional tracheal intubation and to assist during unexpected visualization difficulties of the glottis [6]. Recently a systematic review and meta-analysis clearly confirmed that videolaryngoscopy can offer better glottic views when compared with conventional direct laryngoscopy and therefore is an attractive option for the management of the unexpected difficult airway [7,8]. Thus, videolaryngoscopy has achieved an important role in the management of patients with unanticipated difficult or failed endotracheal intubation. The C-MAC videolaryngoscope is a relatively new device, already investigated in a wide range of clinical studies with promising clinical results [9-13]. However, its suitability in the context of RSI has not yet been studied so far.

### Specific aim of this study

The aim of this study is to investigate the initial success rate of endotracheal intubation with the C-MAC videolaryngoscopy compared with conventional direct laryngoscopy using Macintosh blade in an emergency setting. Additionally, we are going to compare primary and secondary outcome parameters in respect to feasibility and clinical performance of both intubation devices. Furthermore, we aim to evaluate potential technical problems and complications.

In particular we intend to test our primary hypothesis, that RSI using C-MAC is more successful at the first intubation attempt compared with conventional laryngoscopy intubation.

## Methods/Design

### Study design

This study is designed as a prospective, patient-blinded, mono-center, randomized cohort study. This study will be performed at the Emergency Department of the University Hospital Zurich, Zurich, Switzerland.

### Ethical approval

This study has been approved by the local ethic committee of the canton of Zurich (KEK Zurich, reference 2014--356, 31.october 2014) and has been registrated at www.clinicaltrials.gov (identifier NCT02297113).

### Patient population

150 adult patients of both gender, between 18 and 99 years, and requiring emergency RSI at the Emergency Department will be enrolled.

Patients will not be included in this study, if suffering from major maxilla-facial trauma, immobilized cervical spine or if there is any of the following conditions: Indication for awake fiberoptic guided intubation, ongoing Cardio-Pulmonary-Resuscitation (CPR), severe or immediately life-threating injury requiring immediately medical treatment or if patients is already included in any other ongoing clinical trial.

### Sample size calculation

Most available data are from patients with known intubation conditions and in an elective setting. This study focuses on RSI in an emergency setting and is therefore not comparable with other studies. In the emergency setting, the success rate at the initial intubation attempt is the most important clinical outcome parameter. Therefore, we assumed a success rate for the C-MAC intubation of 99% and a success rate for conventional laryngoscopy of 85%. We calculated an estimated total sample size of 144 (72 per group) with a power of 80% and an alpha level of 0.05. Because of potential drop out, we plan to include 150 patients in this study.

### Statistical analysis

Randomized groups will be compared for balance on baseline variables using traditional summary statistics and the standardized difference (STD), defined as the difference in means or proportions divided by the pooled standard deviation. Baseline variables with a STD > 0.20 will be considered imbalanced and will be adjusted for in all analyses. Randomized groups will be compared on outcomes with traditional statistical methods – t-test for normally distributed continuous outcomes, Wilcoxon Rank Sum test for ordinal or non-normal continuous data, and Chi-Square test for nominal variables. Data will be presented as mean with standard deviation, median and interquartile range, or number and percent. A p value < 0.05 is considered to be statistically significant. SPSS statistical software will be used for all analyses.

### Detailed study plan

#### Consent procedure

All patients undergoing emergent RSI at the emergency department will be screened for inclusion in this clinical study. The indication of endotracheal intubation is an exclusively clinical decision and is not affected by this study protocol in any aspect.

Prior to the start of the study each participant has to give their verbal informed consent after he/she was comprehensively informed – verbally on the nature, relevance and impact of the project. Each participant must be informed that the participation in the project is voluntary and that he/she may refuse to participate in the project or withdraw from the project at any time and that withdrawal of consent will not affect his/her subsequent medical treatment. The consent to participate into the research project should be signed and dated by the project leader.

Patients -potentially involved in this study- are critically ill or seriously injured at this juncture. If the detailed enlightenment about this study is not possible, an independent physician not involved in this study will represent the rights of the patients. The study will be explained to the patients and written informed consent will be obtained as fast as possible (patient disposal, a person named therein or a related person). If the patient rejects to participate in this study, the patient will not be intubated according to this study protocol and will be intubated following standard operation procedures at our hospital.

The independent (and not involved in this study) physician will also sign the consent form and confirms, that the patient was adequately informed and agreed to participate into this study or he/she did not visibly express opposition to the research intervention through either verbally or by his or her behavior.

As soon as medically acceptable, but not earlier than the day after surgery/ after extubation and the patient being cognitively capable, the patient will be visited by a researcher and will be enlighted about nature, relevance and impact of this study. Patients will be asked to sign written informed consent. If patient denies inclusion into this study, the patient will be withdrawn from the study.

### Allocation of patients

If fulfilling the in- and exclusion criteria, patient will be randomly assigned to one of two treatment groups:

1. C-MAC videolaryngoscope in appropriate size
2. Conventional endotracheal intubation using Macintosh blade in appropriate size

Randomization (1:1) will be based on computer-generated codes maintained in identical, opaque envelopes that will be opened immediately before randomization.

### Clinical study procedure

All patients transferred to the Emergency Department suffering from severe, acute life-threaten disease or injury. All patients will be monitored including ECG, arterial blood pressure (non-invasive or invasive as appropriate), oxygen saturation (SpO_2_) and will follow standard operating procedures at our department. The bed of the patients will be stored head-up (about 30°). Anesthesia for RSI will be induced with fentanyl 1 to 2 mcg/kg body weight, propofol 1.5 to 3 mg/kg or thiopental 5 mg/kg body weight as clinically appropriate. Succinylcholine 1 mg/kg body weight or rocuronium 1 mg/kg body weight for neuromuscular blockade will be given. Complete muscle relaxation will be confirmed by absence of palpable twitches in response to supra-maximal train-of-four stimulation of the ulnar nerve at the wrist. The tracheal intubation will be performed by one of four highly-skilled researchers, trained in both intubation devices.

The blade-size of both intubation devices (Macintosh or C-MAC) as well as tube size will follow standard procedures at our department and is finally decided by the clinical assessment of the intubating researcher. This judgment is not affected by this study protocol.

The primary endpoint of this study is successful tracheal intubation, confirmed by proofing continuous end-tidal CO_2_ with continuous capnography. Further anesthesia management is independent of this study protocol and will follow standard operation procedure.

### Measurements

#### Primary outcome parameter

1. Successful intubation with first intubation attempt

#### Secondary outcome parameters

2. Time to intubation: defined as time between insertion of the blade into the mouth until detection of end-tidal CO_2,_ measured with integrated timer within the respiration system
3. Total number of intubation attempts
4. Cormack & Lehane score: as determined by intubation performing researcher
5. Inadvertent esophageal intubation
6. Ease of intubation (1–5) [14]: as determined by the intubation performing researcher (very easy, easy, somewhat difficult, difficult, impossible)
7. Complications: diagnosed during and documented immediately after intubation procedure including violations of the teeth, injury/ bleeding of the larynx/ pharynx, and aspiration/regurgitation of gastric content
8. Necessity of using further, alternative airway device for successful intubation (if randomized airway device failed)
9. Maximum drop of saturation
10. Technical problems with the device

### Data collection

All data will be collected by one of the researchers at bedside using the paper-based case report forms (CRF). After finishing recruitment of all patients, the data of the CRF’s will be processed in an electronic database.

## Discussion

In the clinical setting, several studies demonstrated that emergency intubations performed by anesthetists, critical care physicians, and emergency care physicians have had success rates ranging from 97% to 99.3% [15-17]. Unexpected difficulties during laryngoscopy occurred in 5.8% of all patients and poor glottis visualization was encountered in approximately 1–9 % of intubation attempts [18,19]. Several studies suggest, that the C-MAC videolaryngoscope improves glottis visualization, decreases intubation problems and ultimately increases the success rate [20,21]. Especially in the emergency setting, with unknown patient’s airway and stressful environment, intubation tools, which increase the success rate, are urgently needed. However, the benefit of such devices must be clinically assessed by independent researchers, also in the challenging emergency setting.

### Potential study limitations

The most important clinical outcome parameter (success rate with first intubation attempt) is defined as the primary outcome of this study, whereas several studies focus on time to intubation as primary outcome. Although it is easy to measure, clinical relevance seems to be poor. Prolonged and more importantly failed tracheal intubation attempts are associated with negative outcome, as they may cause severe clinical consequences (e.g.iatrogenic oxygen desaturation, aspiration of gastric content or bradycardia) [22-24].

Another potential limitation is that only highly skilled and trained physician will perform intubation and therefore the results cannot be applied to all users. Furthermore, due to ethical considerations, some important emergency patient subpopulations (on-going CPR, major maxilla-facial trauma, immobilized cervical spine, indicated awake fiberoptic guided intubation) will not included in this study.

### Trial status

At the time of submission, the study was actively recruiting patients since November 2014.

## Abbreviations

CPR: Cardio Pulmonary Resuscitation
CRF: Case Report Form
RSI: Rapid Sequence Induction
